# Case of Violent and Long-Continued Head-Ache Removed by Erysipelas on the Face

**Published:** 1829-10-01

**Authors:** 


XVIII.

### WESTMINSTER HOSPITAL.

### CASE OF VIOLENT AND LONG-CONTINUED HEAD-ACHE, &C. DISAPPEARING AFTER ERYSIPELAS OF THE FACE.

Bridget Clarke, æt. 33, admitted June 20th, 1829, under Mr. Guthrie, says her husband infected her two years ago with the venereal disease, and she had a discharge from the vagina and sore throat, as also a small ulcer on the back of her neck. Sometime afterwards her right eye became inflamed and she lost the sight of it. Immediately after this she had a swelling on her forehead which was opened, and from time to time portions of bone came away. There is a cavity now marking the place. She has a small ulcer on her ankle, but without any syphilitic character. Her face is considerably swelled about the eyes, and she says she suffers excessively from a violent pain shooting through her forehead to the back of her head. She complains of loss of power in the right arm, and great pain in both upper extremities, more particularly at the elbows. Her right leg is affected in nearly the same way. The knee-joint is particularly painful. She was ordered on her admission the compound decoction of sarsaparilla with one grain of the oxymuriate of mercury to each dose.

*June 29th.* She does not feel in any degree relieved by her medicine. Pulse 123—tongue rather dry, slightly furred, and yellow in the centre.

℞ Infus Rhei, ℥viij.

Tinct. Card. ʒij.

Carb. Ammon. ℈j. ℳ. ft.

Mist. et cap. cochl. ij. ter in die.

*July 2d.* There is an erysipelatous flush about the upper part of the face. She complains of violent pain in her head and stomach. Bowels not freely opened—tongue furred and dry.

℞ Infus Sennæ, ℥vij.

Pulv. Jalapæ, ʒij.

Potassæ Supertart.

Sodæ Subcarb. āā ʒj. ℳ.

et cap. ℥iss. 2dis. horis.

*July 3d.—a. m.* Her bowels have been freely acted upon. The erysipelas is worse, and she has continual vomiting of a slimy fluid of a dark green colour. Pulse scarcely perceptible. She is ordered two ounces of brandy to be taken slightly diluted in small doses.

*July 4th.* Her pulse is less languid and her face less swelled and inflamed. She feels altogether much better. She had ℥iij. of brandy, and ʒij. of laudanum during the night. Vomiting much less frequent.

℞ Mist. Camph. ℥iss.
Conf. Aromat. gr. x.
Ammon. Subcarb. gr. v. ℳ.
ft. haust. bis terve in die sumend.

*July 5th.* She continues much the same. Complains of great pain in her head and stomach, bowels rather confined. — To continue small doses of brandy.

℞ Hydr. Submur. gr. iv.

Pulv. Jalapæ, c. ʒss.

Pulv. Aromat. gr. iv. ft. pulv.

st. sumend.

*July 8th.* She has continued improving, and has lost the pain in her head.

*July 15th.* Dismissed quite well—all pain in her head and limbs having entirely disappeared.

